# The Short Health Scale: A Valid and Reliable Quality-of-Life Scale for Mainland Chinese Patients with Inflammatory Bowel Disease

**DOI:** 10.1089/pmr.2021.0066

**Published:** 2022-08-18

**Authors:** Jiang-tao Hou, Bin Peng, Shi-jing Zhang, Yong-xin Luo, Yi-ming Chen, Jia-zhong Cai, Yi Wen, Hong Mi, Jian-feng Luo, Xiao-mei Zheng, Si-min Pan, Shi-ying Liu, Xin-lin Chen, Bin Chen

**Affiliations:** ^1^ The First Affiliated Hospital, Guangzhou University of Chinese Medicine, Guangzhou, China.; ^2^ The First Clinical College, Guangzhou University of Chinese Medicine, Guangzhou, China.; ^3^ School of Basic Medical Science, Guangzhou University of Chinese Medicine, Guangzhou, China.; ^4^ Science and Technology Innovation Center, Guangzhou University of Chinese Medicine, Guangzhou, China.; ^5^ Pi-Wei Institute, Guangzhou University of Chinese Medicine, Guangzhou, China.

**Keywords:** inflammatory bowel disease, quality of life, short health scale, translation, validation

## Abstract

**Background:**

The aim of our study was to translate and validate the mainland Chinese version of the short health scale (SHS), a disease-specific quality-of-life (QoL) scale for patients with inflammatory bowel disease (IBD).

**Methods:**

The SHS was translated and validated according to the standard process: a translation and back-translation procedure and a reliability and validation study. Patients with IBD were enrolled, and their QoL was assessed using the SHS, the short inflammatory bowel disease questionnaire (SIBDQ), and the Bristol stool form scale. Reliability (internal consistency reliability, split-half reliability, and test–retest reliability) and validity analyses were performed to evaluate the psychometric characteristics of the SHS. The impacts of different severity of major symptoms on QoL were analyzed by comparing the scores of SHS.

**Results:**

A total of 112 patients with IBD (69 with ulcerative colitis and 43 with Crohn's disease) completed the mainland Chinese version of the SHS, and 34 patients completed the SHS a second time within one to two weeks. Cronbach's alpha value of the SHS was 0.90, and its split-half coefficient was 0.83. Intraclass correlation coefficients of the four items ranged from 0.52 to 0.72. All four items of the SHS were significantly associated with the corresponding domains of the SIBDQ, with correlation coefficients ranging from −0.52 to −0.69 (*p* < 0.001). The results of confirmatory factor analysis indicated a good fit of the one-factor model, with comparative fit index (CFI) = 0.878, normed fit index (NFI) = 0.874, incremental fit index (IFI) = 0.880, and goodness of fit index (GFI) = 0.842. The patients with severe symptoms had higher scores in the SHS than those with no or mild symptoms.

**Conclusions:**

The SHS was simple and quick to be used. The SHS had good validity and reliability and was suitable for evaluating the QoL of patients with IBD in mainland China.

## Background

Inflammatory bowel disease (IBD) is a chronic nonspecific bowel disease of unknown etiology, which includes Crohn's disease (CD) and ulcerative colitis (UC). IBD usually occurs in adolescence and young adulthood and is characterized by a remitting-relapsing disease course.^1^ IBD is found worldwide and poses a great challenge to health care systems around the world. According to epidemiological data, in 2015, the incidence of UC in Europe was as high as 24/100,000, and the incidence of CD was 11.5/100,000.^2^ Ng et al. reported that the incidence of IBD has plateaued in Western countries and risen rapidly in Eastern countries.^3^

The epidemiological data demonstrated that ∼350,000 new cases were diagnosed with IBD between 2005 and 2014 in China. Furthermore, assuming stable prevalence, China would have over 1.5 million cases of IBD by 2025.^4^ IBD had negative effects on patients' physical and mental health and quality of life (QoL).^5^ It is suggested that clinicians should pay more attention to QoL.

Health-related quality of life (HRQoL) has been widely used to assess clinical treatment outcomes in patients with IBD in recent years.^6,7^ HRQoL is a broad, multidimensional concept, covering aspects related to patient perception, experience, daily function, and so on.^8^ The World Health Organization Quality-of-Life (WHOQOL) assessment consists of six domains: physical, psychological, independence, social, environment, and spiritual.^9^ A large number of scales were developed and verified to assess HRQoL in patients with IBD. Fifteen IBD-specific instruments have been developed for patients with IBD.^10^ The most commonly used instruments are the IBD questionnaire (IBDQ),^11^ the short inflammatory bowel disease questionnaire (SIBDQ),^12^ and the short health scale (SHS).^13^ Among them, the IBDQ is the most widely used for patients with IBD and shows promise as a measure of health status for clinical trials in IBD.^14^ The reliability and validity of the mainland Chinese version of the IBDQ have been assessed preliminarily.^15,16^

The SHS is a rapid and specific measurement tool that has been used to assess QoL in patients with IBD in clinical trials and practices.^13,17^ The SHS was developed by Dr Hjortswang in Sweden in 2006.^13^ It is a self-report tool for IBD that uses open-ended questions so that patients can consider aspects that are important to them. To date, the SHS has been translated, validated, and used in Sweden,^13,17^ Norway,^18^ Ireland,^19^ Korea,^20^ and The Netherlands.^21^ The SHS has been proven to be a rapid, valid, and reliable instrument for assessing the QoL of patients with IBD in these countries. Therefore, the purpose of this study was to cross-culturally translate and validate the SHS for Chinese patients with IBD.

## Material and Methods

### Patients

Patients with IBD were enrolled from the First Affiliated Hospital of Guangzhou University of Traditional Chinese Medicine, the First Affiliated Hospital of Sun Yat-sen University, and Shanghai TCM-Integrated Hospital between June and December, 2020. This study was conducted under the Declaration of Helsinki and was approved by the Ethics Committee of the First Affiliated Hospital of Guangzhou University of Traditional Chinese Medicine (No..: ZYYECK [2019]160). Written informed consent was obtained from all enrolled patients.

The inclusion criteria were patients 16–75 years of age with a definite diagnosis of IBD by endoscopy. The exclusion criteria were (1) patients with severe cognitive impairment who could not understand the questionnaire and (2) patients with IBD who refused to participate in the study. Patients had face-to-face interviews with trained researchers. Patients who met the inclusion criteria and were willing to participate were enrolled continuously in the study.

The participants were required to complete the SHS again if they satisfied the following criteria: (1) the patients returned to the hospital within one to two weeks and agreed to complete the questionnaire and (2) the diseases of the patients were relatively stable.

### Translation and back-translation procedure

The researchers contacted Dr Hjortswang, the original author of the SHS, and developed a mainland Chinese version after obtaining Dr Hjortswang's permission. Double forward translation from English to mainland Chinese and backward translation to English were conducted according to Brislin's guidelines for the translation and back-translation method and the standard process for translating instruments.^22,23^ First, two bilingual (mainland Chinese and English) native researchers independently translated the original text into Chinese.

The translation coordinator (one of the authors) integrated and corrected the two mainland Chinese translations to form a first draft of the mainland Chinese version of the SHS. Then, the first draft of the mainland Chinese version of the SHS was back-translated to English by two other bilingual researchers who were not involved in the first translation. The back-translation coordinator (one of the authors) integrated and corrected the two English translations to form the English back-translated version. Finally, the research team coordinated and discussed the differences between the English back-translated version and the original English-translated version of the questionnaire. After language adaptation, the mainland Chinese version of the SHS was finally formed.

### Validation study

The trained researchers explained the purpose of study to patients with IBD. After obtaining informed consent from participants, the researchers asked the patients to complete a self-report case report form (CRF). The CRF contained items about sociodemographic characteristics, the SHS, the SIBDQ, and the Bristol stool form scale (BSFS), major symptoms of IBD. The sociodemographic characteristics included age, sex, education, family history, smoking status, drinking history, diagnosis, and lesion location. If the patients had any questions about the CRF, the researchers would explain the CRF and help to solve the problem. Some participants were also asked to complete the SHS again within one to two weeks after the first survey. All questionnaires were completed using mobile phones or paper in an interview room.

### The short health scale

The SHS is a 4-item self-administered questionnaire that measures the QoL of patients with IBD.^13^ As shown in Supplementary Figure S1, it consisted of four items: symptom, function, worry, and well-being. Each item represents a QoL domain. The items were graded on a 10-cm visual analog scale (VAS). Higher VAS scores indicated worse QoL. The patients with IBD in China were asked to mark the position deemed appropriate on the 10-cm VAS (Supplementary Figure S2).

### The Short Inflammatory Bowel Disease Questionnaire

The SIBDQ is a shortened version of the IBDQ that is responsive to important changes in disease activity.^12^ The SIBDQ consists of ten items, each having seven answer options (all of the time, most of the time, a good bit of the time, some of the time, a little bit of the time, hardly any of the time, and none of the time). The items were clustered into four domains: symptoms, systematic symptoms, social function, and emotional function. The SIBDQ score ranged from 10 (worst QoL) to 70 (best QoL). Higher scores represented higher QoL. The SIBDQ is one of the most common specific scales for measuring QoL in IBD and has been translated and verified in United Kingdom,^12^ the United States,^24^ Germany,^25^ and Spain.^26^

### The Bristol Stool Form Scale

The BSFS is a 7-point scale used extensively in clinical practice and research for assessing stool form.^27^ The usual or most common stool type in the last seven days was assessed using the BSFS. According to the BSFS, the patients were classified into three groups: hard stools (Types 1–2), normal stools (Types 3–5), and loose stools (Types 6–7).^27^ The BSFS was used to assess stability in patients between completing both questionnaires.

### Major symptoms of IBD

A set of questions about major symptoms of IBD was used for assessing disease activity of the participants. These major symptoms were recommended as an efficacy evaluation for colitis in *Development of Clinical Trial of New Drugs of Traditional Chinese medicines* published by the National Medical Product Administration of China.^28^ These symptoms included diarrhea, bloody stools, abdominal pain, and weight loss. Each symptom was rated on a four-point Likert scale from 0 (symptom not present) to 3 (severe). A higher score indicated a more severe symptom. These questions were reported by the participants themselves.

### Statistical methods

All data were input into Microsoft Office Excel 2016, Amos Graphics software (Version 21) and SPSS software version 25.0 (IBM Statistics, Armonk, NY, USA). The Kolmogorov-Smirnov test was used to test the normality of continuous variables. Normally distributed continuous data are expressed as the mean and standard deviation, while nonnormally distributed continuous data are expressed as the median and interquartile range. Categorical variables are presented as percentages (%). The preset *p*-value for significance was 0.05. The correlations between nonnormally distributed variables were analyzed using Spearman's rank correlation coefficient (*r*_s_).

Validity was assessed by correlating both individual SHS domains and total SHS score with corresponding SIBDQ dimensions and total score. We also conducted confirmatory factor analysis (CFA) to validate the structural validity of the instrument as well.

The reliability analysis examined internal consistency reliability, split-half reliability, and test–retest reliability. Cronbach's *α* value was used to assess internal consistency reliability. The reliability was assessed with split-half (odd–even) method based on Spearman-Brown formula. Symptom and worry items were regarded as odd items. Function and well-being items were regarded as even items. Test–retest reliability was evaluated by the intraclass correlation coefficient (ICC) between test–retest scores.

The scores of the SHS domains among the patients with different severity of major symptoms (diarrhea, bloody stools, abdominal pain, and weight loss) were compared using Kruskal-Wallis test, to understand whether those major symptoms affected the QoL among patients with IBD.

## Results

### Patient characteristics

A total of 113 patients were enrolled. One patient was excluded due to incomplete information, and 112 patients were finally included for analysis. A total of 34 participants were included in the test–retest reliability. Demographics and disease-related characteristics for the 112 patients are presented in Table 1. Seventy-two participants (64.3%) were male, with a mean age of 38.9 ± 13.6 years. Among them, 61.6% of the patients had UC, and 38.4% had CD.

**Table 1.** Demographic and Clinical Factors of Patients with Inflammatory Bowel Disease, *n* (%)

Variable	Total (%)	Test–retest (%)
*N*	112	34
Sex		
Female	40 (35.7)	9 (26.5)
Male	72 (64.3)	25 (73.5)
Age (year, mean ± SD)	38.9 ± 13.6	38.0 ± 14.1
Marital status		
Unmarried	39 (34.8)	14 (41.2)
Married	73 (65.2)	20 (58.8)
Past medical history		
No	55 (49.1)	22 (64.7)
Yes	57 (50.9)	12 (35.3)
Family history		
No	111 (99.1)	33 (97.1)
Yes	1 (0.9)	1 (2.9)
Drinking history		
No	86 (76.8)	29 (85.3)
Yes	26 (23.2)	5 (14.7)
Smoking status		
Nonsmoker	72 (64.3)	23 (67.6)
Ex-smoker	30 (26.8)	10 (29.4)
Smoker	10 (8.9)	1 (2.9)
Diarrhea (/day)		
Never	47 (42.0)	16 (47.1)
<3	38 (33.9)	11 (32.4)
3–6	22 (19.6)	5 (14.7)
>6	5 (4.5)	2 (5.9)
Bloody stools		
Never	70 (62.5)	20 (58.8)
Little	34 (30.4)	12 (35.3)
Most	5 (4.5)	0 (0.0)
All	3 (2.7)	2 (5.9)
Abdominal pain		
No	17 (15.2)	5 (14.7)
Mild	55 (49.1)	15 (44.1)
Moderate	25 (22.3)	10 (29.4)
Severe	15 (13.4)	4 (11.8)
Weight loss.		
No	36 (32.1)	9 (26.5)
Mild	25 (22.3)	9 (26.5)
Moderate	22 (19.6)	6 (17.6)
Severe	29 (25.9)	10 (29.4)
BSFS		
Hard (Types 1–2)	3 (2.7)	3 (8.9)
Normal (Types 3–5)	73 (65.2)	25 (73.5)
Loose (Types 6–7)	36 (32.1)	6 (17.6)
Diagnosis		
CD	43 (38.4)	16 (47.1)
UC	69 (61.6)	18 (52.9)
Disease location of UC		
Proctitis	34 (49.3)	11 (61.1)
Left-sided colitis	21 (30.4)	4 (22.2)
Pancolitis	14 (20.3)	3 (16.7)
Disease location of CD		
Colon	7 (16.3)	4 (25.0)
Small bowel	25 (58.2)	10 (62.5)
Colon + small bowel	11 (25.6)	2 (12.5)

BSFS, Bristol stool form scale; CD, Crohn's disease; SD, standard deviation; UC, ulcerative colitis.

### Validity

**FIG. 1.**
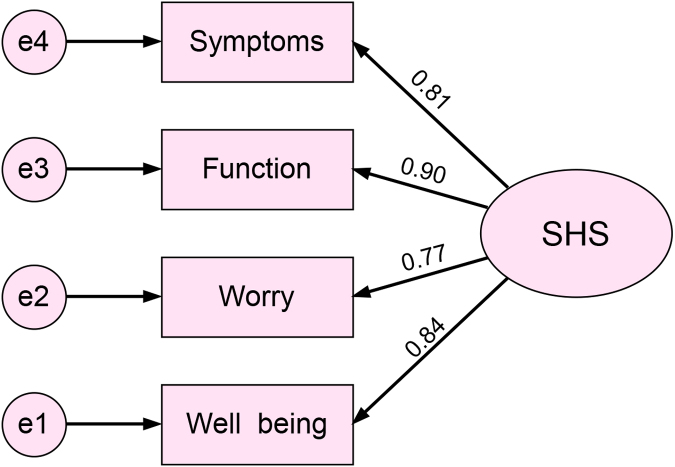
The CFA structure diagram of the SHS. (The number represented the standardized regression coefficient between a particular item and the SHS). CFA, confirmatory factor analysis; SHS, short health scale.

The *r*_s_s of the four SHS items and the corresponding items from the SIBDQ were calculated (Table 2). Among them, the correlation coefficient for SHS general well-being and SIBDQ systemic symptoms was the highest (*r*_s_ = −0.65). The correlation coefficient of symptom items was slightly lower (*r*_s_ = −0.52). The total SHS score was highly correlated with the total SIBDQ score (*r_s_* = −0.69). All correlation coefficients were significant (*p* < 0.001). The CFA results showed the SHS had good structural validity, with all standardized coefficients greater than 0.7 (*p* < 0.05) (Fig. 1) and CFI = 0.878, NFI = 0.874, IFI = 0.880, and GFI = 0.842.

**Table 2.** Spearman Correlation Coefficients (*r*_s_) Between the Short Health Scale and the Short Inflammatory Bowel Disease Questionnaire

The SHS	The SIBDQ	r_s_
Symptom	Symptoms	–0.52
Function	Social function	–0.55
Worry	Emotional function	–0.58
Well-being	Systemic symptoms	–0.65
Total SHS	Total SIBDQ	–0.69

SHS, short health scale; SIBDQ, short inflammatory bowel disease questionnaire.

### Reliability

To evaluate reliability, Cronbach's alpha and ICCs were calculated. Cronbach's alpha for the SHS was 0.90, and the half-split coefficient for the SHS was 0.83, indicating good correlation between items in SHS. A total of 34 IBD patients (16 with CD and 18 with UC) completed the SHS a second time. The subjects completed the questionnaire a second time within 7.4 ± 1.3 days of completing it the first time. Comparison of major symptoms and BSFS reported by participants at two visits is shown in Table 5. All 34 participants reported no significant change of symptoms during follow-up period, suggesting that the disease activity remained stable. Test-retest reliability was evaluated using ICC between test–retest scores (Table 6). The ICC ranged from 0.52 to 0.72.

### Comparison of QoL with different disease type and disease activity

There were no statistically significant differences in the SHS scores of UC and CD patients (Table 3). The association between SHS scores and symptoms is shown in Table 4. All the SHS scores were significantly different for the patients with different symptoms (*p* < 0.05). The patients with severe symptoms had higher scores in the SHS than those with no or mild symptoms.

**Table 3.** The Short Health Scale Scores Between the Patients with Ulcerative Colitis and Crohn's Disease

	Symptom	Function	Worry	Well-being
CD	41.4 (20.0–50.0)^a^	40.1 (20.0–60.0)^a^	55.1 (30.0–80.0)^a^	46.5 (30.0–60.0)^a^
UC	39.4 (20.0–50.0)^a^	43.2 (20.0–60.0)^a^	52.5 (30.0–80.0)^a^	47.0 (30.0–60.0)^a^
*p* ^b^	0.615	0.631	0.494	0.774

^a^ Wilcoxon test.

^b^ The scores were presented as medians and IQRs (25th–75th percentiles).

IQR, interquartile range.

**Table 4.** The Short Health Scale Scores Among the Inflammatory Bowel Disease Patients with Different Symptoms

	Symptom	Function	Worry	Well-being
Diarrhea (/day)				
Never	20.0 (10.0–30.0)	30.0 (15.0–35.0)	40.0 (20.0–50.0)	30.0 (20.0–45.0)
<3	40.0 (30.0–57.5)	35.0 (30.0–57.5)	55.0 (30.0–80.0)	50.0 (30.0–60.0)
3–6	45.0 (30.0–70.0)	50.0 (30.0–77.5)	70.0 (50.0–80.0)	60.0 (42.5–77.5)
>6	100.0 (100.0–100.0)	100.0 (100.0–100.0)	100.0 (100.0–100.0)	100.0 (70.0–100.0)
*p*^a^	<0.001	<0.001	<0.001	0.001
Bloody stools				
Never	30.0 (20.0–50.0)	30.0 (20.0–40.0)	40.0 (20.0–57.5)	35.0 (20.0–50.0)
Little	40.0 (30.0–57.5)	40.0 (30.0–77.5)	75.0 (40.0–80.0)	50.0 (40.0–70.0)
Most	70.0 (60.0–100.0)	80.0 (70.0–100.0)	80.0 (70.0–100.0)	70.0 (50.0–100.0)
All	100.0 (55.0–100.0)	100.0 (65.0–100.0)	100.0 (75.0–100.0)	100.0 (75.0–100.0)
*p*^a^	0.003	<0.001	<0.001	0.001
Abdominal pain				
No	30.0 (10.0–40.0)	20.0 (10.0–30.0)	20.0 (10.0–40.0)	20.0 (10.0–30.0)
Mild	30.0 (20.0–50.0)	30.0 (20.0–50.0)	50.0 (30.0–80.0)	40.0 (30.0–50.0)
Moderate	30.0 (30.0–50.0)	40.0 (30.0–50.0)	60.0 (40.0–80.0)	50.0 (40.0–70.0)
Severe	60.0 (40.0–80.0)	70.0 (45.0–90.0)	80.0 (65.0–100.0)	80.0 (55.0–100.0)
*p*^a^	0.038	<0.001	<0.001	0.001
Weight loss				
No	25.0 (10.0–32.5)	25.0 (10.0–30.0)	30.0 (20.0–52.5)	30.0 (17.5–50.0)
Mild	30.0 (20.0–50.0)	30.0 (20.0–40.0)	40.0 (30.0–50.0)	30.0 (30.0–50.0)
Moderate	40.0 (30.0–60.0)	45.0 (30.0–60.0)	70.0 (32.5–80.0)	50.0 (40.0–60.0)
Severe	50.0 (30.0–80.0)	70.0 (30.0–90.0)	80.0 (50.0–100.0)	70.0 (50.0–80.0)
*p*^a^	<0.001	<0.001	<0.001	0.001
BSFS^b^				
Hard	20.0 (10.0–30.0)^c^	23.3 (10.0–30.0)^c^	56.7 (20.0–70.0)^c^	43.3 (10.0–70.0)^c^
Normal	31.9 (20.0–40.0)	32.7 (20.0–40.0)	44.5 (20.0–60.0)	38.9 (20.0–50.0)
Loose	58.6 (40.0–80.0)	62.8 (30.0–95.0)	71.4 (50.0–100.0)	63.1 (50.0–80.0)
*p*^a^	<0.001	<0.001	<0.001	0.001

^a^ Kruskal-Wallis test.

^b^ For BSFS (Bristol stool form scale), Hard: Types 1–2; Normal: Types 3–5; Loose: Types 6–7.

^c^ The scores were presented as medians and IQRs (25th–75th percentiles).

**Table 5.** Change of Symptoms Over a Follow-Up Period (Visit 1 and Visit 2)

	Visit 1 (*n*)	Visit 2 (*n*)	*p*
Diarrhea (/day)			0.661
Never	16	16	
<3	11	11	
3–6	5	7	
>6	2	0	
Bloody stools			0.185
Never	20	18	
Little	12	13	
Most	0	3	
All	2	0	
Abdominal pain			0.460
No	5	10	
Mild	15	14	
Moderate	10	6	
Severe	4	4	
Weight loss			0.565
No	9	13	
Mild	9	6	
Moderate	6	8	
Severe	10	7	
BSFS			0.910
Hard (Types 1–2)	3	3	
Normal (Types 3–5)	25	23	
Loose (Types 6–7)	6	8	

^a^ Wilcoxon test.

**Table 6.** Test–Retest Reliability for 34 Inflammatory Bowel Disease Patients

Variable	Visit 1 SHS	Visit 2 SHS	*p*	ICC (95% CI)
Symptom	30.0 (20.0–52.5)	40.0 (20.0–52.5)	<0.001	0.71 (0.49–0.84)
Function	30.0 (20.0–52.5)	30.0 (20.0–60.0)	<0.001	0.52 (0.22–0.73)
Worry	50.0 (30.0–80.0)	50.0 (30.0–80.0)	<0.001	0.72 (0.51–0.85)
Well–being	40.0 (30.0–62.5)	40.0 (30.0–62.5)	<0.001	0.53 (0.25–0.73)

The scores were presented as medians and IQRs (25th–75th percentiles).

CI, confidence interval; ICC, intraclass correlation coefficient.

## Discussion

The SHS is a simple and quick scale to be used in mainland Chinese patients with IBD, and it has good operability. The patients with IBD were asked to mark the appropriate position on a 10 cm VAS. A total of 112 valid questionnaires were collected. Moreover, most patients completed the mainland Chinese version of the SHS within 1 minute. And our patients appeared to have little difficulty during the investigation. The results were also consistent with those from other countries.^6,19–21^ Accordingly, the SHS is a feasible tool for Chinese patients and clinicians.

The mainland Chinese version of the SHS had good structural validity. We found good associations for most items were more than 0.55, except for the symptom item. The symptom item showed a slightly lower correlation with the corresponding SIBDQ item (0.52). The results were consistent with a study using the SIBDQ as a comparator in Dutch-speaking patients, whose scores ranged from 0.403 to 0.828.^21^ McDermott et al. translated the SHS into English for English-speaking patients in Ireland. They used the IBDQ as a comparator, and they reported that the correlation coefficients of all SHS items ranged from 0.662 to 0.737. The correlation coefficients in their study were higher than ours.^19^ The reason may be related to the selection of different comparator instruments.

Reliability was assessed by internal consistency reliability, split-half reliability, and test–retest reliability. These tests indicated that the SHS was reliable. Cronbach's alpha values above 0.70 were considered satisfactory.^29^

Similarly, the SHS had good test-retest reliability, as assessed with the test–retest method in 34 patients after one to two weeks. The ICC values of the symptom and worry items were both greater than 0.7, which indicated acceptable test–retest reliability.^30^ Our results showed that the ICC of the SHS domains was between 0.52 and 0.72, which were lower than those reported by Edel et al. Edel et al. reported that test–retest correlations ranged from 0.64 to 0.91 for SHS domains, with a test–retest time interval of 2 weeks and 38 IBD patients.^19^ The reason for this is unclear, but it may be that subtle changes occur in patients' attitudes to stable disease over time, which requires further investigation.

There were some limitations for this research. (1) The sample size of the study was small. The data of patients with IBD should be collected from larger areas in future studies. (2) Disease activity was assessed using the following symptoms, including diarrhea, bloody stools, stool type, abdominal pain, and weight loss. These symptoms do not fully represent disease activity. (3) Only 34 patients completed the SHS a second time. (4) In addition, information on medication was not collected for patients with IBD.

## Conclusions

The SHS is a simple and quick scale for clinical research and practice in mainland Chinese patients with IBD. This study confirmed that the mainland Chinese version of the SHS had good validity and reliability and was suitable for evaluating the QoL of Chinese patients with IBD.

## Supplementary Material

Supplemental data

Supplemental data

## Data Availability

X.-L.C. and B.P. had full access to all data in the study and take responsibility for the integrity of data and the accuracy of data analysis. The datasets used and analyzed in this study are available from the corresponding author on reasonable request.

## Supplementary Material

Figure S1. The English version of the SHS

Figure S2. The Chinese version of the SHS

## Abbreviations Used

BSFS: Bristol stool form scale
CD: Crohn's disease
CFA: confirmatory factor analysis
CI: confidence interval
CRF: case report form
HRQoL: health-related quality of life
IBD: inflammatory bowel disease
IBDQ: IBD questionnaire
ICC: intraclass correlation coefficient
IQR: interquartile range
QoL: quality of life
SHS: short health scale
SIBDQ: short inflammatory bowel disease questionnaire
UC: ulcerative colitis
VAS: visual analog scale
