# Supplementary figures and images for: The Short Health Scale: A Valid and Reliable Quality-of-Life Scale for Mainland Chinese Patients with Inflammatory Bowel Disease
Source: Palliat Med Rep. 2022 Aug 18;3(1):154–61. doi: 10.1089/pmr.2021.0066 (PMC9438447; doi:10.1089/pmr.2021.0066)

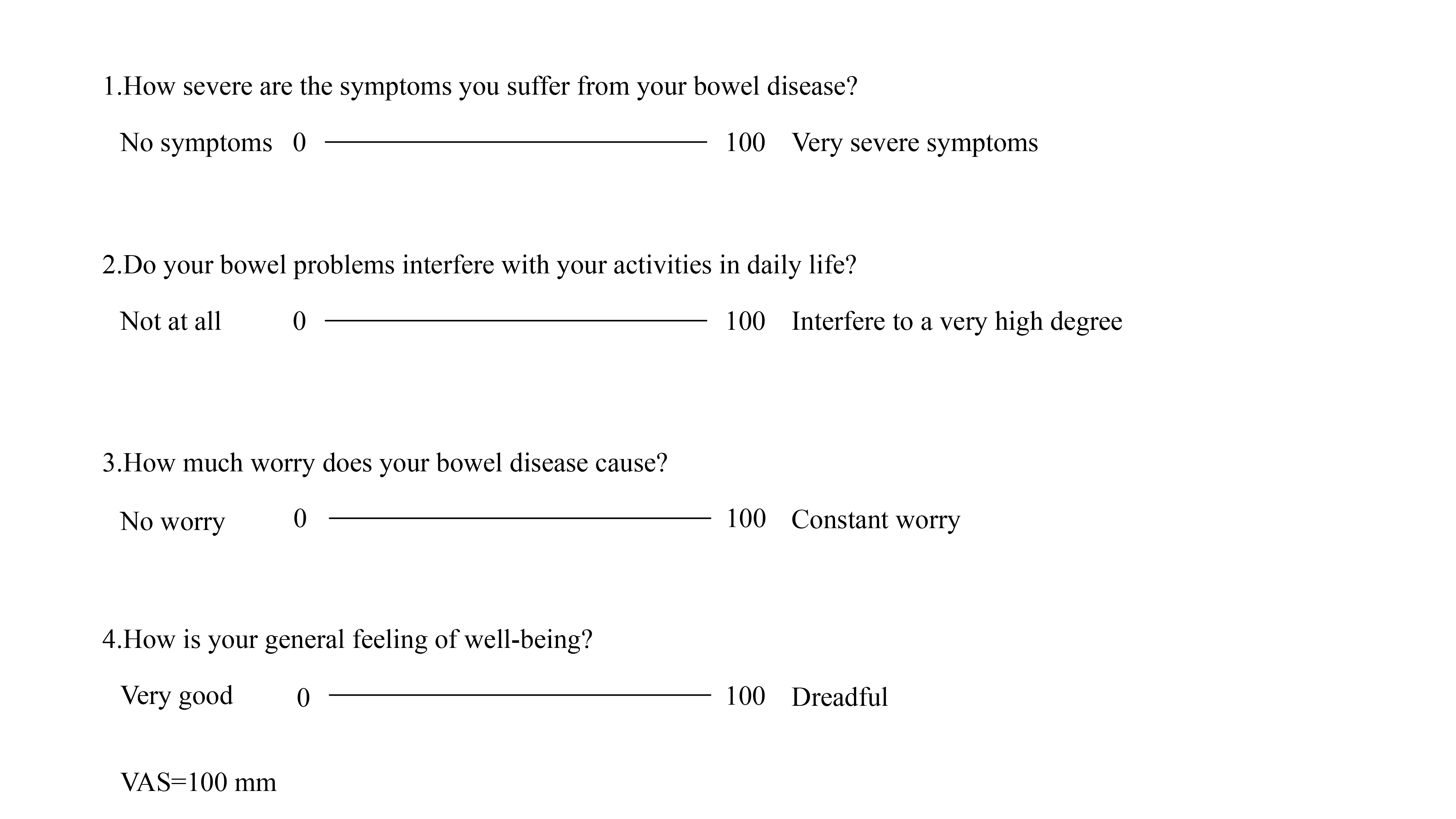

Supplement: Supplemental data [file Supp_FigS1.tif]

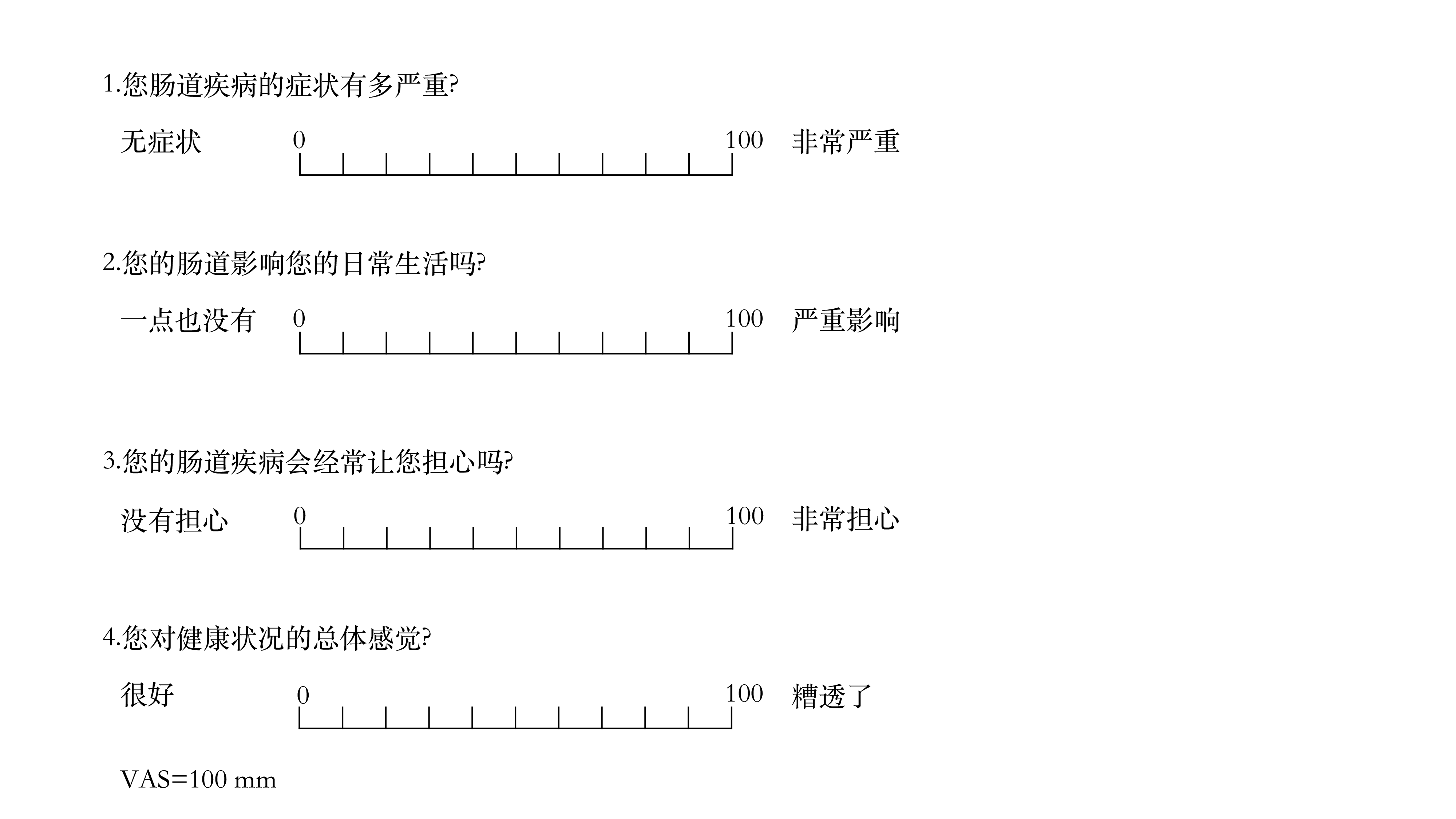

Supplement: Supplemental data [file Supp_FigS2.tif]
